# Erratum

**DOI:** 10.1111/jdi.12470

**Published:** 2016-03-02

The authors would like to draw the reader's attention to the error in the following article.

Kaku K, Kiyosue A, Ono Y, Shiraiwa T, Kaneko S, Nishijima K, Bosch‐Traberg H, Seino Y. Liraglutide is effective and well tolerated in combination with an oral antidiabetic drug in Japanese patients with type 2 diabetes: A randomized, 52‐week, open‐label, parallel‐group trial. *J Diabetes Investig* 2016; 7: 76–84.

Figure 1 (participant flow during trial) lists, in the Additional OAD group, the number of withdrawals for ‘Other’ reasons as *n* = 11; this should have read *n* = 3.

The authors apologize for the error and any confusion it may have caused.

